# An extraocular electrical stimulation approach to slow down the progression of retinal degeneration in an animal model

**DOI:** 10.1038/s41598-023-40547-1

**Published:** 2023-09-23

**Authors:** Alejandra Gonzalez Calle, Javad Paknahad, Dimitrios Pollalis, Pragya Kosta, Biju Thomas, Ben Yi Tew, Bodour Salhia, Stan Louie, Gianluca Lazzi, Mark Humayun

**Affiliations:** 1 Department of Ophthalmology, USC Roski Eye Institute, Keck School of Medicine, University of Southern California, Los Angeles, CA 90033 USA; 2 USC Ginsburg Institute for Biomedical Therapeutics, University of Southern California, Los Angeles, CA 90033 USA; 3 USC Institute for Technology and Medical Systems Innovation, Los Angeles, CA 90033 USA; 4 USC Department of Translational Genomics, Keck School of Medicine, University of Southern California, Los Angeles, CA 90033 USA; 5 USC Mann School of Pharmacy and Pharmaceutical Sciences, University of Southern California, Los Angeles, CA 90089 USA

**Keywords:** Neuroscience, Engineering

## Abstract

Retinal diseases such as retinitis pigmentosa (RP) and age-related macular degeneration (AMD) are characterized by unrelenting neuronal death. However, electrical stimulation has been shown to induce neuroprotective changes in the retina capable of slowing down the progression of retinal blindness. In this work, a multi-scale computational model and modeling platform were used to design electrical stimulation strategies to better target the bipolar cells (BCs), that along with photoreceptors are affected at the early stage of retinal degenerative diseases. Our computational findings revealed that biphasic stimulus pulses of long pulse duration could decrease the activation threshold of BCs, and the differential stimulus threshold between ganglion cells (RGCs) and BCs, offering the potential of targeting the BCs during the early phase of degeneration. In vivo experiments were performed to evaluate the electrode placement and parameters found to target bipolar cells and evaluate the safety and efficacy of the treatment. Results indicate that the proposed transcorneal Electrical Stimulation (TES) strategy can attenuate retinal degeneration in a Royal College of Surgeon (RCS) rodent model, offering the potential to translate this work to clinical practice.

## Introduction

Retinal dystrophies such as retinitis pigmentosa (RP), age-related macular degeneration (AMD), and primary open-angle glaucoma (POAG), is characterized by unrelenting neuronal death (photoreceptor loss in RP and AMD and ganglion cell loss in POAG)^1^. Several mechanisms have been identified as potential causes of neuronal death occurring in these diseases, such as genetic mutations in RP, dysfunction in lipid metabolism and inflammation in AMD, and elevated intraocular pressure in POAG^1–3^. Although treatments to ameliorate these conditions exist, in many cases blindness continues to progress. Therefore, approaches to prevent blindness and slow down the progression of retinal degeneration at the very early disease stages would make a big impact on the lives of patients with retinal diseases.

Different treatment strategies, proposed to prevent retinal disease progression include gene therapy, cell therapy, retinal prostheses, and transcorneal electrical stimulation (TES). Gene and stem cell therapies that aim to target the molecular pathogenesis associated with AMD and RP have shown significant progress over the years. However this approach has limitations in that the clinical outcome of these approaches targets very specific populations^4^. Retinal prosthesis is the only current treatment for patients with end-stage retinal degeneration^5^; unfortunately, patients in the early stages of diagnoses with retinal blindness diseases cannot benefit from prosthetic devices as they still have meaningful visual function.

TES treatment can preserve the outer nuclear layer and enhancet the electroretinography function (ERG) after 6 weeks of stimulation^6,7^ in an in vivo model. Studies have also shown how electrical stimulation can benefit neuronal preservation such as via modulation in neurotropic factors after a session of TES^8^, and how different stimulation parameters can be used in order to target different cell types^9^. However, the results from these TES studies have yielded mixed results or nominal benefits in vision and therefore systematic research is needed to determine the most effective electrical stimulation approach for selective induction of stimulation in retinal neurons.

Herein, both multi-scale computational model and modeling platform were used for designing electrical stimulation strategies, including the placements of electrodes and stimulus waveforms, to better target the bipolar cells, that are affected first at the early stage of retinal degenerative diseases such as retinitis pigmentosa. These computational findings were then verified by conducting in vivo experiments in the Royal College of Surgeons (RCS) rats to test the proposed electrical stimulation approaches from the modeling framework and evaluate the progression of their retinal degeneration.

## Materials and methods

### Computational modeling

**Figure 1:**
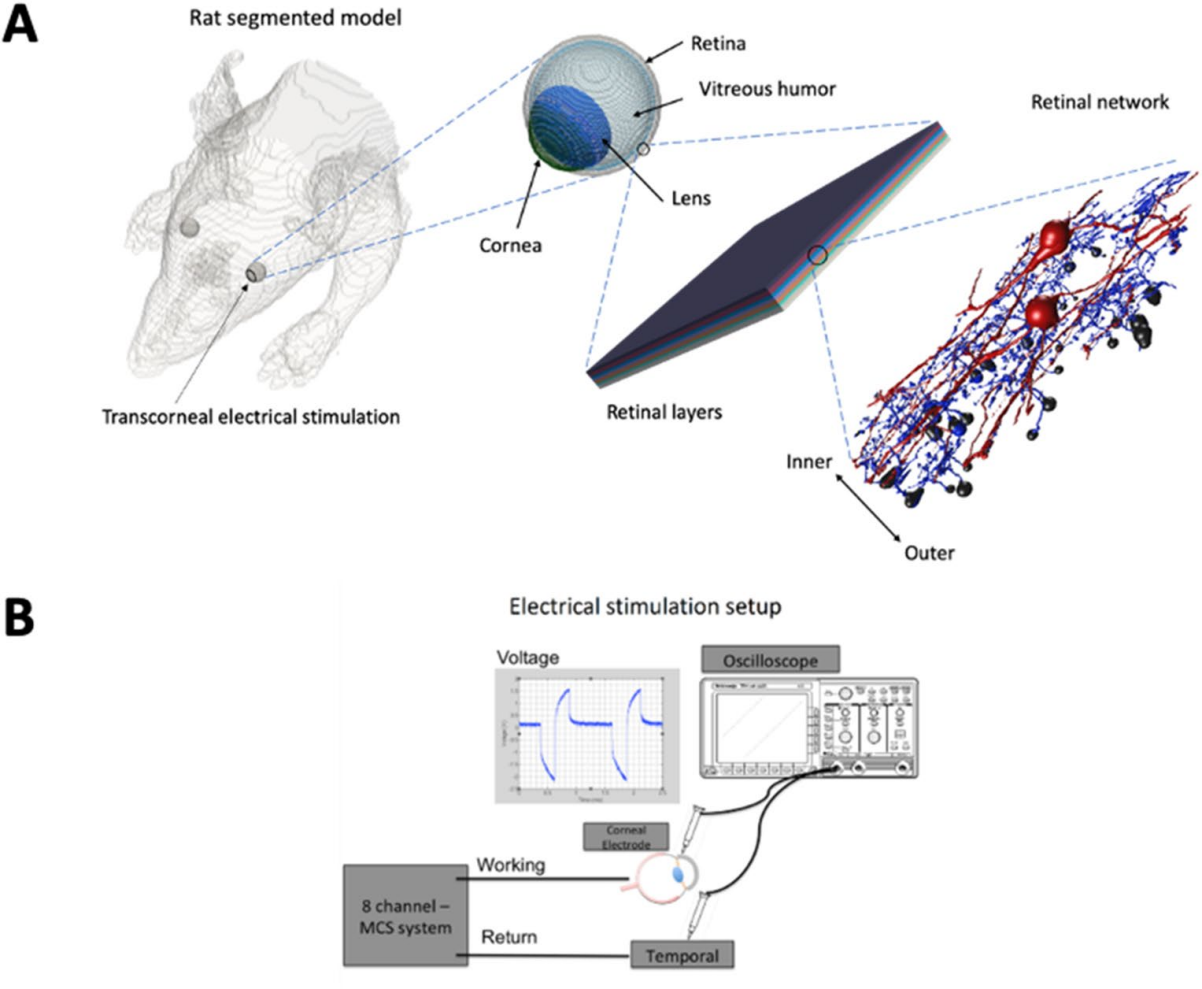
(**A**) Multi-scale Admittance Method (AM)/NEURON computational platform is capable of constructing a large-scale rat voxel model, representing fine details of the eye, simulating different retinal layers based on their resistivity values, and cellular-level modeling of the retinal network, including retinal ganglion cells (RGCs) and bipolar cells (BCs). This modeling platform can predict the electric fields generated inside the retinal tissue due to transcorneal electrical stimulation (TES) and determine the activation threshold of RGCs and BCs to different electrical stimulation parameters. (**B**) Schematic representation of the setup used for TES of the RCS rats, MCS: multi-channel stimulation.

Our group has leveraged computational modeling to investigate the stimulation paradigms for efficient electrical stimulation of retinal cells as well as to examine the behavior of healthy and degenerated retinal cells^10–13^. We have developed an Admittance Method AM/NEURON multi-scale computational modeling platform that has been deployed to: (1) predict the electric fields generated inside the retina tissue due to TES; (2) couple the extracellular voltages to biophysically and morphologically realistic models of RGCs and BCs; (3) determine the activation threshold of these cells to different electrical stimulation parameters^14–16^. This multi-scale modeling framework is capable of constructing a large-scale segmented rat model including the finer ocular structures as well as micro-scale modeling of retinal layers and connectome as shown in Fig. 1A. A computational model of retinal cells, spiking diffuse BCs (DB4) and A2 RGCs, was developed and validated with experimental data from the literature^17–20^ and used to capture their response under the considered external electrical stimulation to identify the greatest non-invasive electrical stimulation approach to reduce the activation threshold of BCs. Further, the impact of electrode configurations and placements on the excitation threshold of retinal neurons was evaluated as well.

The rat voxel model utilized in this study was adapted from the developed full mouse model described in^21^, with adjustments made to reflect the size of RCS rats. Additionally, the resolution of the original voxel model was increased to incorporate the fine structure of the detailed eye into the model as shown in Fig. 1A. The resistivity of various tissues utilized in the model is obtained from^22^ as detailed in Table 1. The minimum resolution of the multi-resolution rat voxel AM model was set to 166 µm and a maximum merged element size of 64 voxels was applied. The final computational model is composed of approximately 400 million computational cells. In our modeling approach, we simplified the representation of the degenerated retinal layers in Fig. 1A for computational efficiency, assuming a homogeneous retinal layer with an average resistivity of 1.5 Ω m (Table 1), which has been verified to be a reasonable approximation. The stimulation waveforms of symmetric charge-balanced biphasic and monophasic pulses over a range of pulse durations from 0.1 to 25 ms were tested. The resulting extracellular voltages induced in the tissue were applied to the multi-compartment models of retinal cells in the simulation platform, based on NEURON^23^, which is integrated into the computational multi-scale simulation package.

**Table 1:** Resistivity of various tissues in the rat voxel model of Fig. 1A.

Tissue name	Resistivity [Ω m]
Muscle	4.82
Skin	5000
Fat	64.61
Bone marrow	762.95
Bone cancellous	12.67
Brain	25.72
Vitreous humor	0.666
Retina	1.5
Lens	3.15
Cornea	2.4

To model the spiking BCs, we utilized the morphology of BCs extracted from^24^. The resulting multi-compartmental model of BCs incorporates a finely compartmentalized structure with a non-uniform distribution of ionic channels at each section. The model investigates the response characteristics of BCs to a range of electrical stimulation parameters. Specifically, the DB4-BCs compartmental model includes several ionic currents, such as sodium, slow and fast potassium, L-type and T-type calcium, and hyperpolarization-activated current^25^. The membrane capacitance is set to 1 μF/cm^2^, and an intracellular resistivity of 100 Ω cm is chosen for the model. The maximum ionic conductance values for each section of this subtype of BCs are listed in Table 2. For additional information regarding the remaining parameters and variables, please refer to^12,24^.

**Table 2:** The ion channel densities of the model spiking BCs [mS/cm^2^].

	Soma	Dendrite	Axon	Presynaptic terminal	Terminal
g_Na_	–	–	1000	–	–
g_Kslow_	0.6	2.4	–	–	–
g_Kfast_	–	–	2	–	–
g_caL_	–	–	–	1	–
g_caT_	1	1	–	–	–
g_HCN_	–	–	–	3.25	–
g_l_	0.033	0.033	0.033	0.033	0.033

The membrane properties and dynamics of ionic channels of A2-RGCs were calibrated based on single-compartment models of these cells, and the intracellular responses were validated using experimentally recorded signals of the RGC subtype^26^. The morphological parameters of the A2 cell were obtained by extracting the SWC file from the NeuroMorpho database^27^. The model of the cell included a precise representation of axonal properties, including the axon initial segment, which serves as the site of spike initiation, sodium channel band (SOCB), axon hillock (AH), narrow segment (NS), and the distal axon (DA)^18^ Supplementary Figure 1. The total axon length and soma diameter were set to 1000 µm and 20 µm, respectively. The dendritic field size was defined as 320 µm. The densities of ionic channels in each region of the cells, as well as the kinetics of ionic currents and the voltage-dependent gating variables, were based on our previous modeling work as provided in Table 3. The membrane capacitance of the cells was set to 1 µF/cm^2^, while the intracellular resistivity was set to 110 Ω cm. A uniform distribution of leaky current was assumed throughout the cells. The reversal potential and conductance values of the leak ion channel were − 60 mV and 0.05 mS/cm^2^, respectively. Further details can be found in our previous studies^11,15,18,19^.

**Table 3:** The ion channel densities of the A2-RGCs model [S/cm^2^].

	Soma	Dendrite	AH	SOCB	NS	DA
g_Na_	0.35	0.1	0.8	2.4	0.9	0.8
g_K_	0.12	0.05	0.6	0.8	0.6	0.6
g_K,A_	3*g_K_	3*g_K_	3*gK	3*gK	3*gK	3*gK
g_K,Ca_	0.004*g_K_	0.004*g_K_	–	–	–	–
g_Ca_	0.137	0.05	–	–	–	–
g_h_	0	0	–	–	–	–
g_T_	0.004	0	–	–	–	–

### Transcorneal electrical stimulation in vivo

In order to study the effect on the progressive and rapid retinal degeneration seen in the Royal College of Surgeons (RCS) rats, animals starting at the age of 20 days through 60 days old were used to assess the effects of transcorneal electrical stimulation. The RCS rats have an inherited retinal degenerative disorder secondary to a MERTK mutation (gene encoding for Proto-oncogene tyrosine-protein kinase enzyme) making them a great model to study the preservation and restoration of vision^28^. All animals were maintained on a daily 12 h light/day cycle before experiments. Animal experiments were in accordance with Animal Research: Reporting of In vivo Experiments (ARRIVE) guidelines and approved by the Institutional Animal Care and Use Committee (IACUC) of the University of Southern California (USC) that is in adherence to the National Institutes of Health (NIH) Guidelines for the Care and Use of Laboratory Animals, and The Association for Research in Vision and Ophthalmology (ARVO) Statement for the Use of Animals in Ophthalmic and Vision Research.

Stimulation was performed in only the right eye (OD) of each animal (n = 18) for 2 h, once a week, beginning at postnatal day 20–21 (P20-P21). Animals received TES at P21, P28, P35, P42, P49, and P56. A 3 mm in diameter platinum-stimulating ring electrode was placed on the cornea (right eye) and used as the stimulating electrode (Fig. 1B). The ground electrode was placed temporally on the stimulated eye. For every stimulation session, RCS rats were anesthetized using ketamine (80 mg/kg) and xylazine (8 mg/mL) via intraperitoneal (IP) injection, and their body temperature was regulated and maintained at 37 °C with a water heating pad. Heart rate and respiration were monitored throughout the experiment. Animals were euthanized and ocular tissue was collected at P60 for histological analysis.

### Superior colliculus recording

Electrophysiological recordings of electrically evoked responses in the superior colliculus (SC) were performed in anesthetized animals following our previously established methodology^29^. SC experiments were performed to find the threshold retinal response to transcorneal electrical stimulation. The eyes of the RCS rats (P45) were covered with an opaque cap to prevent rod saturation during the surgical preparation. After unilateral parietal craniotomy, the SC was exposed by cortical suction. A custom-made tungsten microelectrode was inserted superficially into the SC and advanced 200 microns below the surface and targeting the central area of the SC. To verify electrode placement, baseline responses were recorded using a full field light flash (Grass model PS 33 Photic stimulator, W. Warwick, RI, USA) onto the contralateral eye at an intensity of 0.19 log cd/m^2^.

After baseline responses were recorded, both stimulating and ground electrodes were placed on the right eye (OD) of all animals as explained above. Biphasic, cathodic-first pulses were used. A pulse width of 10 ms was kept constant throughout the experiments and pulse amplitude was incremented in steps of 5 µA until an electrically evoked response was observed in the superior colliculus as compared to the response to the full field light flash stimulation (positive control).

### Fundus autofluorescence imaging

Fundus autofluorescence (FAF) imaging is a common method used to evaluate the progression of RPE and retina health in patients with dystrophies like age-related macular degeneration and retinitis pigmentosa^30^. A commercially available cSLO (SPECTRALIS HRA + OCT, Heidelberg, Germany) was used to perform FAF imaging on 18 P60 RCS rats (20 μA n = 5, 50 μA n = 5, 100 μA n = 8). In brief, pupils were dilated using 2.5% phenylephrine hydrochloride and 1% tropicamide. Rats were anesthetized using ketamine (80 mg/kg) and xylazine (8 mg/kg) via intraperitoneal (IP) injection. A baseline imaging was done at P21 and animals with the pre-existing retina or optic nerve defects were eliminated from the study. The cornea was kept moist with repeated application of normal saline during all in vivo imaging. Two FAF images of different sensitivities were taken of each eye having the optic nerve in the center as a hallmark at P58. For FAF image analysis, FIJI (NIH, USA) was used. Briefly, the area of interest was selected using the freehand select tool, excluding the optic nerve and vessels. Then the selected area of interest was thresholded and its area was measured.

### Histology

Harvested eyes were collected at the end of the study, and were Histological analysis was performed in 10 animals (20 μA n = 3, 50 μA n = 3, 100 μA n = 4). Both the control and stimulated eyes of each animal were fixed in Davidson’s fixative for 24 h and embedded into paraffin. Eyecup sections were then stained with hematoxylin and eosin and the outer nuclear layer (ONL) was evaluated using Aperio CS2 (Leica Microsystems, Germany). H&E-stained eyecups were scanned, and photoreceptor count (ONL) was acquired from a 1 mm section located 1 mm away from the optic nerve in the inferior area of the retina. Photoreceptor counts were acquired from both treated and non-treated eyes.

### Statistical analysis

Unless otherwise described, all values were reported as mean ± standard deviation (SD). Statistical analysis used GraphPad Prism, where statistical differences were measured with Student’s *t-test,* where statistical significance was established a priori with an alpha of 0.05.

## Results

### Computational modeling: electrode configurations

**Figure 2:**
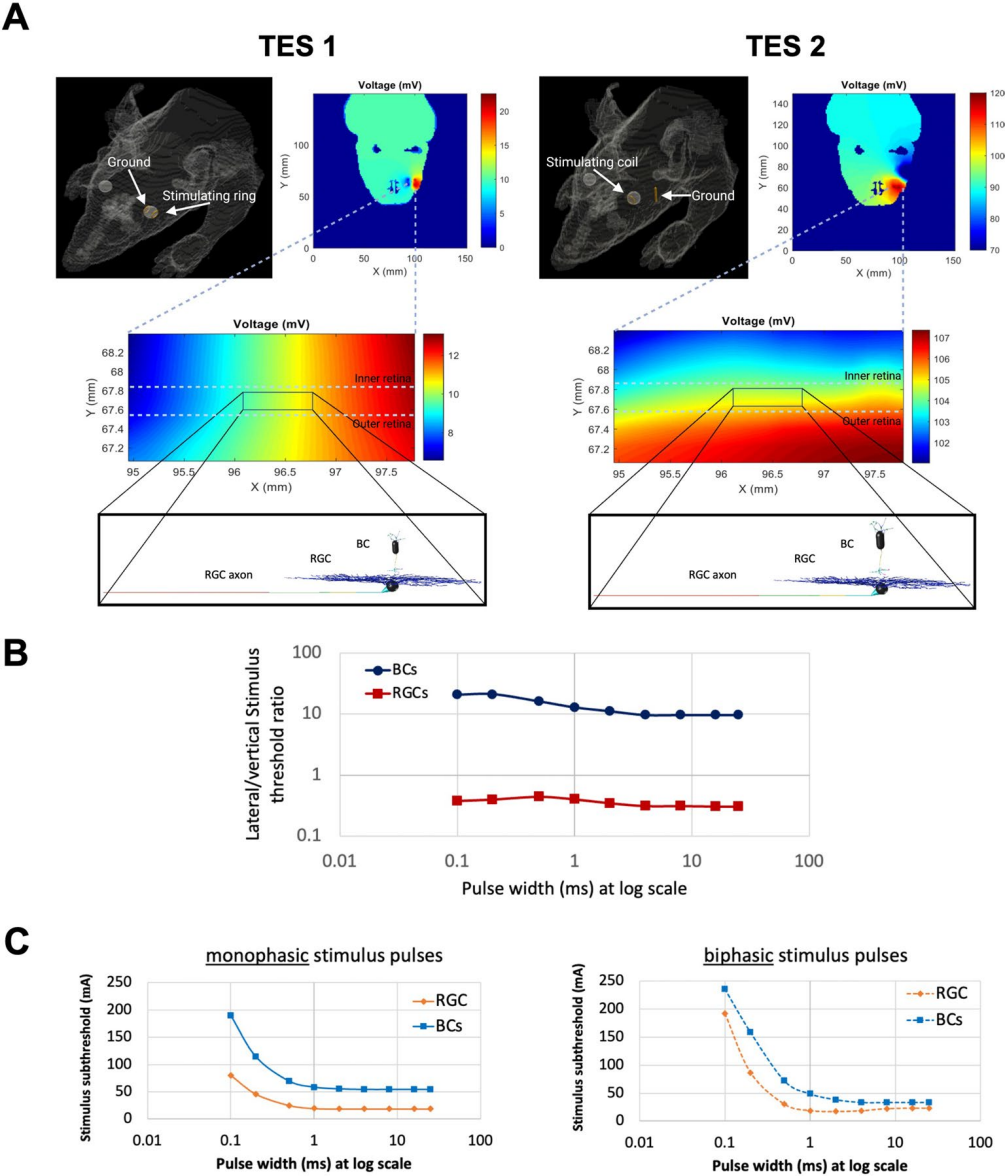
(**A**) Two transcorneal electrical stimulation setups are simulated: TES1 and TES2. For TES1, a stimulating ring and a ground ring electrode are positioned toward the temporal and the nasal side of the eyeball, respectively. For TES2, a stimulating ring electrode is placed on the cornea, and a needle ground electrode is placed on the head towards the temporal side. The results are shown for a slice of the model and the color map represents the voltage distribution (mV) in the tissue, including the retina. These extracellular voltages were extracted from the central retina (the box in the figure) and applied to each compartment in multi-compartment models of RGCs and BCs. For TES1, the voltage gradient is generated laterally along the RGCs axon (lateral stimulation), while the TES2 is maximizing the induced field along the BCs axons and the retinal thickness (vertical stimulation) (**B**) For both RGCs and BCs, the ratio of stimulation thresholds for TES1 to TES2 is computed for a range of stimulus pulse durations. BCs have a tenfold higher stimulation threshold for TES1 than TES2, whereas, RGCs have a lower stimulation threshold for TES1 than TES2. (**C**) The strength-duration curves for a range of pulse durations from 0.1 to 25 ms for both monophasic and biphasic stimulus pulses. Results suggest that long biphasic pulse durations can augment the chance for the excitations of BCs and reduce the differential stimulation threshold of RGCs and BCs.

Using the developed models of retinal bipolar and ganglion cells, we have utilized a multi-scale computational modeling framework to determine the best electrode placements and electrical stimulation parameters to focalize the induced electric fields to the eye and effectively activate and target the outer retinal neurons such as bipolar cells. Figure 2A represents the two placements and configurations of the stimulating and ground electrodes for TES of the rat model. In the first transcorneal electric stimulation setup (TES 1), the stimulating ring is placed on the temporal side, and the return electrode is on the nasal region. In the second transcorneal electrical stimulation setup (TES 2), a needle ground electrode is placed on the temporal site and the stimulating ring is placed on the cornea. Resulting extracellular voltages induced in the retina tissue from the AM model were applied to multi-compartment models of neurons using NEURON software^23^ to analyze the neuronal responses of the developed RGCs and BCs. Morphological sizes of retinal neurons and particularly bipolar cells as one of the smallest neurons in the nervous system are in the order of 10 µm to 400 µm. Therefore, to save the computation time of NEURON simulations and increase the spatial resolution of the resulting voltages, we analyzed only a section of the central retina. We used a 3D interpolation function to achieve a finer resolution of the AM voltage distribution and apply it as an extracellular voltage to each compartment of multi-compartment models of RGCs and BCs. As illustrated, the two stimulation setups lead to different generated voltage gradients along the two retinal neurons (Fig. 2A). To slow down the progression of retinal blindness in patients at the early stage of the disease, BCs should be the main target of electrical stimulation as they are affected first compared to RGCs. Therefore, the TES setup with the maximum induced electric field along the axon of BCs is desired, leading to the enhanced likelihood of voltage-gated ion channels opening and reduced threshold of BCs. As shown in Fig. 2A, TES 2 setup results in the largest generated voltage gradient along the retinal thickness and further focalizes the voltage distribution to the eye.

To better examine the influence of electrode placement and configuration on the response of retinal neurons, we further compared the stimulation threshold of RGCs and BCs for electrical stimulation in TES 1 and TES 2. The stimulation thresholds of both RGCs and spIking BCs were determined by identifying the lowest stimulus current amplitude that elicits action potentials. The ratio of retinal neuron stimulation thresholds of TES1 to TES2 setups for a range of stimulus pulse durations is shown in Fig. 2B. While the TES1 setup can significantly reduce the stimulation threshold of RGCs, it appears to be ineffective for electrically stimulating BCs as the BCs threshold is almost 10-fold higher than the RGCs threshold. However, the TES 2 stimulation strategy allows us to target BCs better as the stimulation threshold of this cell type is significantly reduced compared to TES 1. The lower stimulation threshold of BCs observed with TES 2 compared to TES1 can be attributed to the higher electric field generated along the bipolar cell axon in TES 2, coupled with a higher density of sodium channels in the axon. The increased electric field strength in TES 2 facilitated more efficient activation of bipolar cells, leading to a lower stimulation threshold. The mechanisms of electrical stimulation and the concept of activating function have been extensively discussed in the literature^31–33^.

### Computational modeling: pulse width and frequency

Utilizing the TES 2 setup, we applied both monophasic and biphasic stimulus waveforms of various pulse durations and computed the stimulation thresholds of the cells. The strength-duration curves of both stimulus waveforms are plotted in Fig. 2C. Our initial computational findings show that the activation threshold of bipolar cells is higher relative to retinal ganglion cells. This arises from the smaller diameter of axons in BCs compared to RGCs. However, our modeling reveals that using biphasic stimulus pulses with long pulse duration can reduce the activation threshold of BCs as well as the differential stimulus threshold between the RGCs and BCs (Fig. 2C). Congruent to our simulation results, experimental data from the literature indicate that long pulse duration increases the likelihood of activation of BCs and therefore indirect activation of RGCs. Further, Walston et al. have shown experimentally using the wholemount mouse retina that the response of ON-type BCs is desensitized as the stimulation frequency increases^34^. The response of BCs was shown to be stable at a low stimulation frequency of 6 Hz. Therefore, the symmetric charge-balanced cathodic-first biphasic stimulation with a pulse duration of 10 ms and stimulus frequency of 6 Hz was selected for the initial in-vivo experiments.

### Shannon safety curve and superior colliculus stimulation

**Figure 3:**
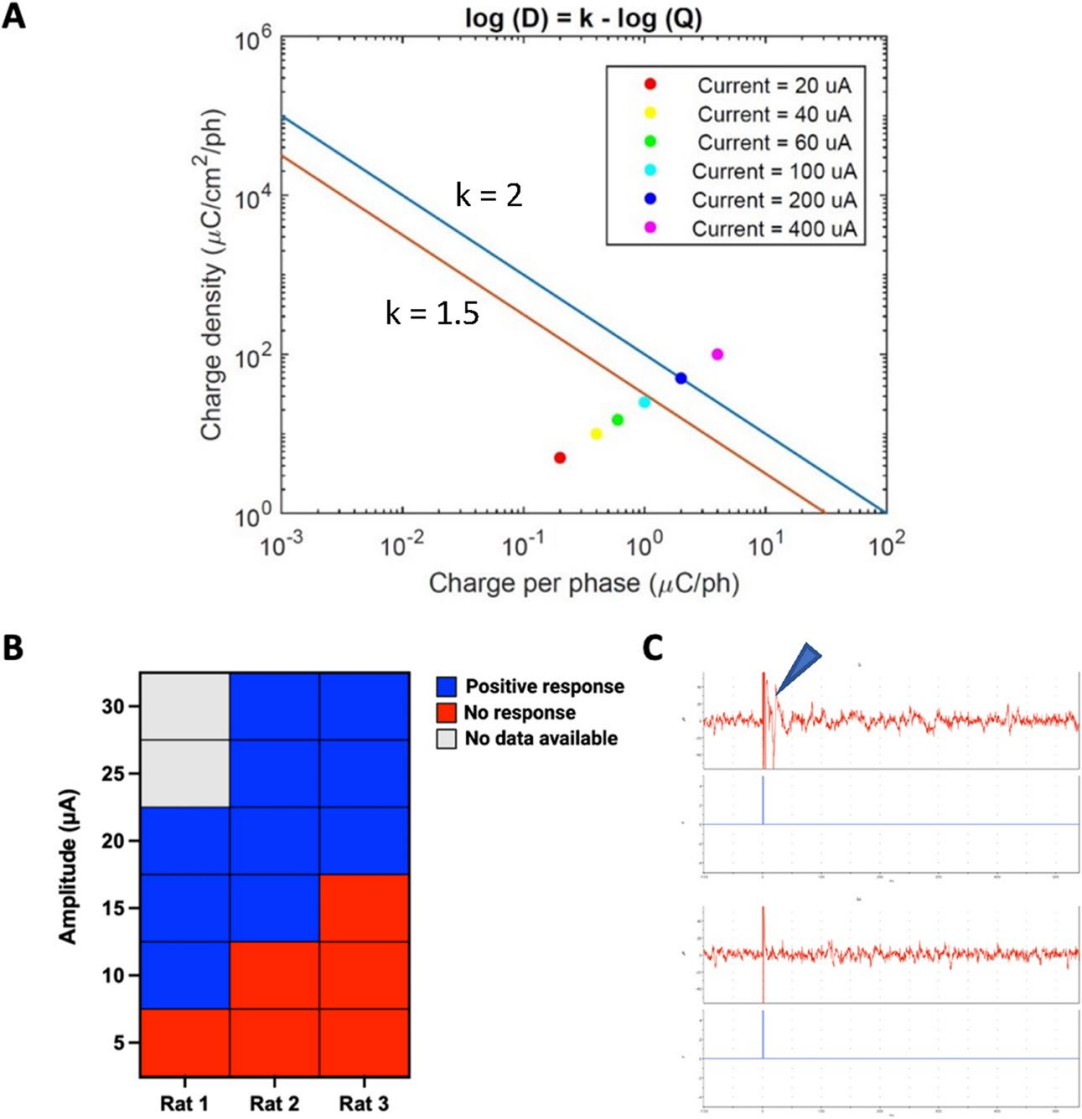
(**A**) A Shannon safety curve (charge density per phase versus charge per phase for a range of current amplitude) indicated that using current amplitudes up to 100 µA (light blue dot) allows the safe delivery of electrical stimulation to the eye. The estimation of the boundary between safe and unsafe injected charge into the tissue can be approximated using the equation log(D) = k − log(Q), where D represents the charge density, Q represents the charge per phase, and k is the parameter determining the damage boundary. The blue line corresponds to k = 2, indicating the region where tissue damage is observed. Conversely, the red line represents k = 1.5, indicating the threshold below which no damage was observed. (**B**) Electrically evoked responses recorded from the superior colliculus of three RCS rats showed a threshold response of 5, 10, and 15 μA respectively. (**C**) Superior colliculus electrophysiology. Top—representative graphs of electrically evoked SC activity (arrow), bottom—absence of apparent light evoked SC responses in the above animal.

To ensure the safe delivery of electrical stimulation through the cornea, we carried out the safety analysis according to Shannon safety theory to find the maximum current amplitude that could be safely used^35^. A platinum-stimulating ring electrode with an inner diameter of 3 mm and a wire diameter of 270 µm was used for this analysis. We plotted charge density per phase versus charge per phase for a range of current amplitude from 20 to 400 µA as shown in Fig. 3A. Results indicate that using current amplitudes up to 100 µA (light blue dot) allows the safe delivery of electrical stimulation to the eye.

The lower threshold used during the stimulation was found through the electrically evoked responses recorded from the superior colliculus. Three different RCS rats had a threshold response of 5, 10, and 15uA respectively (Fig. 3B and C). Based on the superior colliculus and Shannon curve results the window of stimulation for the experiments reported went from 20 to 100 μA.

### Assessment of retinal degeneration

**Figure 4:**
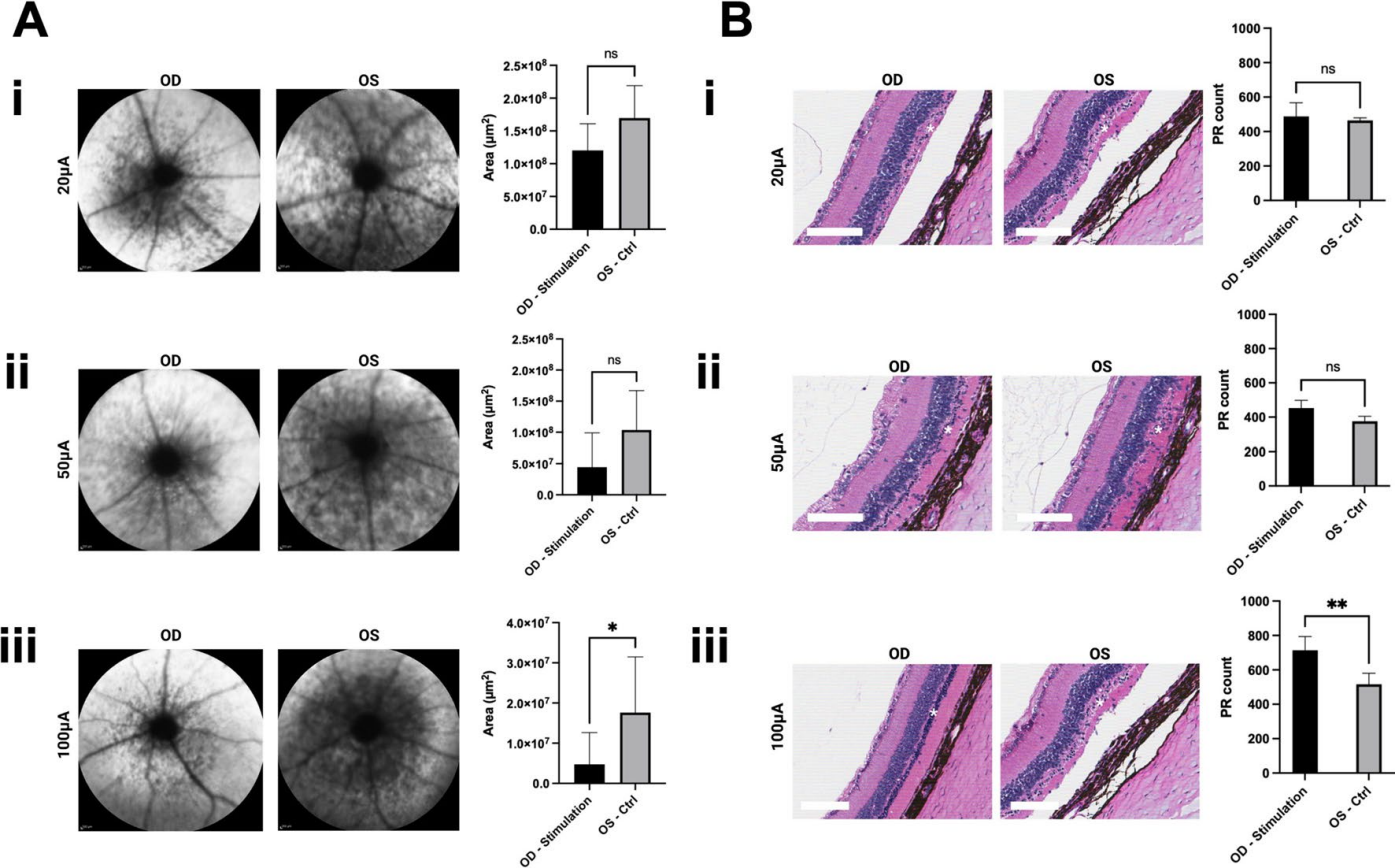
(**A**) Area analysis of hypopigmentation in the degenerated retina in autofluorescence fundoscopy images (20 μA group n = 5, 50 μA group n = 5, 100 μA group n = 5) showed no significant difference between the stimulated and control eye in 20 μA (**i**) and 50 μA (**ii**) groups. A 73% reduction in the area of retinal degeneration was observed in the stimulated eye compared to the control one in the 100 μA group (**iii**). (**B**) Photoreceptor count analysis in H&E-stained retinas (20 μA group n = 3, 50 μA group n = 3, 100 μA group n = 4) showed no significant difference between the stimulated and control eye in 20 μA (**i**) and 50 μA (**ii**) groups. A 38.3% PR count increase was observed in the stimulated eye compared to the control one in the 100 μA group (**iii**). (*< p 0.05, **< p 0.01, scale bar, 100μm).

The progression of the disease was evaluated by monitoring the area of retina hypopigmentation using FAF imaging. It was observed that for the 20 μA and 50 μΑ groups, although the stimulated eye followed a pattern of less hypopigmented area compared to the control fellow eye, the difference was not statistically significant (p-value 0.12 and 0.15 respectively) (Fig. 4Ai and ii). On the other hand, when stimulation at 100 μA was given to the rats, a 73% reduction of the area of retinal degeneration was observed in the treated eye compared to the untreated one (p-value 0.038) (Fig. 4Aiii).

Furthermore, histological analysis also supported the improvement of the retinal degeneration of the 100 μΑ group. Although PR count analysis showed no statistical significance between the stimulated and control eye of both 20 μA and 50 μA groups (p-value 0.64 and 0.065 respectively) (Fig. 4Bi and ii), a 38.3% PR count statistically significant increase was observed at the 100 μA-stimulated group compared to the control ones (p-value 0.008) (Fig. 4Biii).

## Conclusion and discussion

Given the fact that controlled electrical stimulation can induce neuroprotective effects in neurons and in particular retinal cells^36–38^, we have, for the first time, developed a multi-scale computational method capable of analyzing the response of retinal neurons to transcorneal electrical stimulation. We deployed this modeling framework to design electrical stimulation setups for effectively inducing electric fields in the retina for therapeutic approaches. We further designed electrical stimulus waveforms to reduce the stimulation threshold of BCs that are influenced first at the early stage of retinal degeneration to induce protection to the damaged cells and therefore slow down the progression of retinal blindness^39,40^. We found that retinal bipolar cells are more sensitive to long stimulus pulses of TES, offering the intriguing potential to induce epigenetic and neuroprotective changes to primarily affected bipolar cells.

In order to optimize the therapeutic effect and mitigate potential discomfort for patients undergoing TES, it is crucial to carefully consider the thresholds of both BCs and RGCs. While the primary objective of our study was to reduce the stimulation threshold of BCs, the direct activation of RGCs through TES can lead to prolonged phosphene activations, flashing sensations, and therefore patient discomfort. To address this concern, we aimed to minimize the threshold difference between BCs and RGCs, thereby reducing the potential for direct RGCs activation while still eliciting BCs responses. This balanced approach ensures a more favorable therapeutic outcome by maximizing the likelihood of BCs activation while minimizing adverse effects caused by excessive RGCs stimulation. By taking into account both BC and RGC thresholds, our study aims to improve the efficacy and safety of TES as a potential treatment modality for retinal disorders.

Furthermore, we particularly focused on modeling spiking bipolar cells in this study. The decision was based on several considerations. While it is true that most BC types are generally considered non-spiking in nature, their responses to electrical stimulation manifest primarily as graded potentials. These graded potentials exhibit varying amplitudes in relation to the stimulus strength, as demonstrated in our recent publication including the experimental data^13^. However, determining the activation thresholds for non-spiking BCs becomes challenging without incorporating the entire retinal network into the model. The complex interactions within the network can significantly influence the generation and propagation of graded potentials. It is also worth noting that there are subtypes of cone bipolar cells as well that exhibit spiking behavior due to the presence of voltage-gated sodium channels^41,42^. Given the current scope of our computational modeling study, which aimed to investigate the effects of transcorneal electrical stimulation on BCs and RGCs, we specifically focused on modeling spiking BCs to better understand their activation thresholds and further design stimulation parameters to decrease the differential stimulus threshold of RGCs and BCs.

It is important to note that the computational model aimed to provide relative comparisons of stimulus thresholds of RGCs and BCs for different stimulation waveforms and electrode placements rather than an exact quantitative match with the experimental data. The difference in absolute values of stimulation thresholds between the computation model and measurements can be attributed to several factors. Firstly, our computational model specifically focused on modeling RGCs and BCs in the central region of the retina, where the electrode was not in close proximity (Fig. 2A). As a result, the electric field gradient in the central retina was lower compared to the peripheral region, leading to higher stimulation thresholds in the computational study. In contrast, the experimental measurements encompassed the entire retina, including cells in the peripheral region where the stimulation threshold is lower due to the close proximits to the stimulating ring electrode. Furthermore, the findings of this study indicated that increasing the stimulation amplitude can preserve outer retinal neurons and slow down the progression of retinal degeneration (Fig. 4). Therefore, further increasing the stimulation amplitude could potentially enhance the therapeutic effect of TES through enhanced neuronal activities. However, due to safety concerns, the stimulation current amplitude was not further increased beyond 100 µA in this study as shown in Fig. 3A. These factors should be considered when interpreting the results and comparing the computational and experimental findings.

FAF imaging indicated a slower progression of retinal degeneration in the treated eye compared to the non-treated eye and preservation of the photoreceptor layer relative to the non treated eye. These preliminary findings have proven that this computational modeling approach helped predict the stimulation parameters for effectively inducing electric fields in the retina and stimulating retinal cells preferentially.

Different retinal degenerative diseases affect different retinal layers. This computational modeling approach will allow us to evaluate and customize electrical stimulation treatments for different conditions. Further animal testing needs to be carried out in different disease models to confirm the outcome of this current study and other parameters specific to other conditions like POAG.

It has been demonstrated that electrical stimulation of the retina cells can lead to transcriptomic changes that are indicative of neuroprotective changes, including downregulation of proapoptotic genes such as Bax and upregulation of prosurvival genes such as brain-derived neurotrophic factor (BDNF). The overarching hypothesis of this study is that neuroepigenetic and chromatin remodeling of the retina induced through controlled electrical stimulation is a key molecular determinant of neuroprotection and could prove to be pivotal for the treatment of retinal degenerative diseases^43^.

We can conclude that the novelty work presented in this report includes: (1) a large-scale rat segmented model including the finer structures of the eye as well as micro-scale modeling of retinal layers and connectome was developed to design electrical stimulation parameters for slowing down the progression of RP disease in a rat model; (2) maximization of the induced electric fields in the retina were accomplished by utilizing the electrode placements from the multi-scale AM-NEURON computational modeling framework; (3) our in vivo experiments allowed us to analyze the response of retinal neurons to transcorneal electrical stimulation; (4) out results have proven the proposed TES strategy effective in preserving the outer layer of the RCS rat model retina (Fig. S1).

### Supplementary Information

Supplementary Figure S1.

## Data Availability

The datasets used and/or analyzed during the current study are available from the corresponding author on reasonable request.

## References

[1] Wang AL, Knight DK, Vu TT, Mehta MC (2019). Retinitis Pigmentosa: Review of current treatment. Int. Ophthalmol. Clin..

[2] Zhang Q (2016). Retinitis pigmentosa: Progress and perspective. Asia Pac. J. Ophthalmol..

[3] Hamel C (2006). Retinitis pigmentosa. Orphanet J. Rare Dis..

[4] Daiger SP, Sullivan LS, Bowne SJ (2013). Genes and mutations causing retinitis pigmentosa. Clin. Genet..

[5] Lan Y (2020). Retina–electrode interface properties and vision restoration by two generations of retinal prostheses in one patient—one in each eye. J. Neural Eng..

[6] Morimoto T (2007). Transcorneal electrical stimulation promotes the survival of photoreceptors and preserves retinal function in royal college of surgeons rats. Investig. Ophthalmol. Vis. Sci..

[7] Morimoto T (2005). Transcorneal electrical stimulation rescues axotomized retinal ganglion cells by activating endogenous retinal igf-1 system. Investig. Ophthalmol. Vis. Sci..

[8] Fu L, Lo ACY, Lai JSM (2015). The role of electrical stimulation therapy in ophthalmic diseases. Graefe's Arch. For Clin. Exp. Ophthalmol..

[9] Sehic A (2016). Electrical stimulation as a means for improving vision. Am. J. Pathol..

[10] Cela, C. J. A multiresolution admittance method for large-scale bioelectromagnetic interactions. Ph.D. thesis, North Carolina State University (2010).

[11] Paknahad J, Humayun M, Lazzi G (2022). Selective activation of retinal ganglion cell subtypes through targeted electrical stimulation parameters. IEEE Trans. Neural Syst. Rehabil. Eng..

[12] Paknahad J, Kosta P, Bouteiller JMC, Humayun MS, Lazzi G (2021). Mechanisms underlying activation of retinal bipolar cells through targeted electrical stimulation: a computational study. J. Neural Eng..

[13] Paknahad, J., Kosta, P., Iseri, E., Farzad, S., Bouteiller, J. M. C., Humayun, M. S., & Lazzi, G. Modeling ON cone bipolar cells for electrical stimulation. In *2021 43rd Annual International Conference of the IEEE Engineering in Medicine & Biology Society (EMBC)* 6547–6550. (IEEE, 2021). 10.1109/EMBC46164.2021.9629884 PMC8754156 34892609

[14] Paknahad J, Kosta P, Bouteiller JMC, Humayun MS, Lazzi G (2021). The sensitivity of retinal bipolar cells response to long stimulus pulse durations in epiretinal prostheses. Investig. Ophthalmol. Vis. Sci..

[15] Paknahad J, Loizos K, Yue L, Humayun MS, Lazzi G (2021). Color and cellular selectivity of retinal ganglion cell subtypes through frequency modulation of electrical stimulation. Sci. Rep..

[16] Iseri, E., Kosta, P., Paknahad, J., Bouteiller, J. M. C., & Lazzi, G. A computational model simulates light-evoked responses in the retinal cone pathway. In *2021 43rd Annual International Conference of the IEEE Engineering in Medicine & Biology Society (EMBC)*, 4482–4486 (IEEE, 2021). 10.1109/EMBC46164.2021.9630642 PMC10578446 34892214

[17] Kosta P, Iseri E, Loizos K, Paknahad J, Pfeiffer RL, Sigulinsky CL, Anderson JR, Jones BW, Lazzi G (2021). Model-based comparison of current flow in rod bipolar cells of healthy and early-stage degenerated retina. Exp. Eye Res..

[18] Paknahad J, Loizos K, Humayun M, Lazzi G (2020). Targeted stimulation of retinal ganglion cells in epiretinal prostheses: A multiscale computational study. IEEE Trans. Neural Syst. Rehabil. Eng..

[19] Paknahad, J., Loizos, K., Humayun, M., & Lazzi, G. Responsiveness of retinal ganglion cells through frequency modulation of electrical stimulation: A computational modeling study. In *2020 42nd Annual International Conference of the IEEE Engineering in Medicine & Biology Society (EMBC)*, 3393–3398 (IEEE, 2020). 10.1109/EMBC44109.2020.9176125 PMC7997733 33018732

[20] Kosta P, Loizos K, Lazzi G (2020). Stimulus waveform design for decreasing charge and increasing stimulation selectivity in retinal prostheses. Healthc. Technol. Lett..

[21] Mendes BM, de Almeida IG, Trindade BM, Fonseca TCF, de Campos TPR (2017). Development of a mouse computational model for MCNPx based on Digimouse(r) images and dosimetric assays. Braz. J. Pharm. Sci..

[22] Italian National Research Council - Institute for Applied Physics (CNR-IFAC). An Internet resource for the calculation of the Dielectric Properties of Body Tissues in the frequency range 10 Hz–100 GHz. http://niremf.ifac.cnr.it/tissprop/.

[23] Carnevale NT, Hines ML (2006). The NEURON Book.

[24] Rattay F, Bassereh H, Stiennon I (2018). Compartment models for the electrical stimulation of retinal bipolar cells. PLoS ONE.

[25] Werginz P, Rattay F (2016). The impact of calcium current reversal on neurotransmitter release in the electrically stimulated retina. JNE..

[26] Qin W, Hadjinicolaou A, Grayden DB, Meffin H, Burkitt AN, Ibbotson MR, Kameneva T (2017). Single-compartment models of retinal ganglion cells with different electrophysiologies. Network.

[27] Ascoli GA (2006). Mobilizing the base of neuroscience data: The case of neuronal morphologies. Nat. Rev. Neurosci..

[28] Strauss O, Stumpff F, Mergler S, Wienrich M, Wiederholt M (1998). The Royal College of Surgeons rat: an animal model for inherited retinal degeneration with a still unknown genetic defect. Acta Anat..

[29] Thomas BB, Seiler MJ, Sadda SR, Aramant RB (2004). Superior colliculus responses to light-preserved by transplantation in a slow degeneration rat model. Exp. Eye Res..

[30] Bubis E, Sher I, Skaat A (2019). Blue autofluorescence fundus imaging for monitoring retinal degeneration in royal college of surgeons rats. Transl Vis Sci Technol..

[31] Rattay F (1999). The basic mechanism for the electrical stimulation of the nervous system. Neuroscience.

[32] Rattay, F. Analysis of models for external stimulation of axons. In *IEEE Transactions on Biomedical Engineering*, vol. BME-33, no. 10, 974–977 10.1109/TBME.1986.325670 (1986). 10.1109/TBME.1986.325670 3770787

[33] Werginz P, Wang BY, Chen ZC, Palanker D (2020). On optimal coupling of the 'electronic photoreceptors' into the degenerate retina. J. Neural Eng..

[34] Walston ST, Chow RH, Weiland JD (2018). Direct measurement of bipolar cell responses to electrical stimulation in wholemount mouse retina. J. Neural Eng..

[35] Shannon RV (1992). A model of safe levels for electrical stimulation. IEEE Trans. Biomed. Eng..

[36] Miyake K-I, Yoshida M, Inoue Y, Hata Y (2007). Neuroprotective effect of transcorneal electrical stimulation on the acute phase of optic nerve injury. Investig. Ophthalmol. Vis. Sci..

[37] Yu H (2020). Noninvasive electrical stimulation improves photoreceptor survival and retinal function in mice with inherited photoreceptor degeneration. Investig. Ophthalmol. Vis. Sci..

[38] Henrich-Noack P, Sergeeva EG, Sabel BA (2017). Non-invasive electrical brain stimulation: From acute to late-stage treatment of central nervous system damage. Neural Regen. Res..

[39] Xie J (2010). Modeling and percept of transcorneal electrical stimulation in humans. IEEE Trans. Biomed. Eng..

[40] Potts AM, Inoue J, Buffum D (1968). The electrically evoked response of the visual system (eer). Investig. Ophthalmol..

[41] Pan ZH, Hu HJ (2000). Voltage-dependent Na(+) currents in mammalian retinal cone bipolar cells. J. Neurophysiol..

[42] Cui J, Pan Z (2008). Two types of cone bipolar cells express voltage-gated Na channels in the rat retina. Vis. Neurosci..

[43] Willmann G, Schaferhoff K, Fischer MD, Arango-Gonzalez B, Bolz S, Naycheva L, Rock T, Bonin M, Bartz-Schmidt KU, Zrenner E, Schatz A, Gekeler F (2011). Gene expression profiling of the retina after transcorneal electrical stimulation in wild-type Brown Norway rats. Investig. Ophthalmol. Vis. Sci..

