# Author Correction: An extraocular electrical stimulation approach to slow down the progression of retinal degeneration in an animal model

**DOI:** 10.1038/s41598-023-45262-5

**Published:** 2023-10-23

**Authors:** Alejandra Gonzalez Calle, Javad Paknahad, Dimitrios Pollalis, Pragya Kosta, Biju Thomas, Ben Yi Tew, Bodour Salhia, Stan Louie, Gianluca Lazzi, Mark Humayun

**Affiliations:** 1 Department of Ophthalmology, USC Roski Eye Institute, Keck School of Medicine, University of Southern California, Los Angeles, CA 90033 USA; 2 USC Ginsburg Institute for Biomedical Therapeutics, University of Southern California, Los Angeles, CA 90033 USA; 3 USC Institute for Technology and Medical Systems Innovation, Los Angeles, CA 90033 USA; 4 USC Department of Translational Genomics, Keck School of Medicine, University of Southern California, Los Angeles, CA 90033 USA; 5 USC Mann School of Pharmacy and Pharmaceutical Sciences, University of Southern California, Los Angeles, CA 90089 USA

Correction to: *Scientific Reports* https://doi.org/10.1038/s41598-023-40547-1, published online 23 September 2023

The Acknowledgments section in the original version of this Article was omitted. The Acknowledgments section now reads:

“This research was based upon work supported by grants from the National Science Foundation under Grant Nos 1933394, 2121164; and supported/partially supported by the USC Center for Neuronal Longevity (#PG1033624); Unrestricted Grant to the Department of Ophthalmology from Research to Prevent Blindness, New York, NY; Dr. Allen and Charlotte Ginsburg Institute for Biomedical Therapeutics; Dennis and Michele Slivinski; The USC Roski Eye Institute; Dr. Ramani Nathan; and The Retina Research Foundation's Gertrude D. Pyron Award. No funding sponsors were involved in the study design, collection, analysis, interpretation of data, writing of the report, or decision to submit the article for publication. The following authors have a provisional patent (US Patent App. 17/685186) through the University of Southern California (USC): Mark S. Humayun, Gianluca Lazzi, Bodour Salhia, Ben Yi Tew, Javad Paknahad, Alejandra Gonzalez-Calle.”

The original Article has been corrected.

