# Supplementary material for: An extraocular electrical stimulation approach to slow down the progression of retinal degeneration in an animal model
Source: Sci Rep. 2023 Sep 23;13:15924. doi: 10.1038/s41598-023-40547-1 (PMC10517961; doi:10.1038/s41598-023-40547-1)
Supplement: Supplementary file 1 — Supplementary Figure S1. [file 41598_2023_40547_MOESM1_ESM.docx]

**Figure S1.** The membrane voltages at soma, dendrites, SOCB, distal axon (at the edge) in response to TES1 and TES2. SOCB is the site of spike initiation, and the spike is generated at the edge of the RGCs axon with great latency, indicating that the site of spike initiation for TES (TES1 & TES2) is similar to focal electrical stimulation of cells, and the spike is not initiated at the RGCs terminal.

|  |  |
| --- | --- |
